# Assessment of Static Balance with and Without Cognitive Dual Task in Children with Haemophilia: A Cross-Sectional Study

**DOI:** 10.3390/jfmk11010067

**Published:** 2026-02-06

**Authors:** Ana Chimeno-Hernández, Pilar Alberola-Zorrilla, Jorge Martín Campos, Juan J. Carrasco, Sofía Pérez-Alenda, Daniel Sánchez Zuriaga, Marta Aguilar-Rodríguez

**Affiliations:** 1 Physiotherapy in Motion. Multispecialty Research Group (PTinMOTION), Department of Physiotherapy, University of València, 46010 Valencia, Spain; ana.chimeno@uv.es (A.C.-H.); juan.j.carrasco@uv.es (J.J.C.); marta.aguilar@uv.es (M.A.-R.); 2 Research Group on the Clinical Anatomy of the Musculoskeletal System (GIACAL), Department of Anatomy and Human Embryology, Universitat de València, 46010 Valencia, Spain; pilar.alberola@uv.es (P.A.-Z.); daniel.sanchez@uv.es (D.S.Z.); 3 Asociación Regional Murciana de Hemofilia, 30120 El Palmar, Spain; jorgemartin.fisio@gmail.com; 4 Intelligent Data Analysis Laboratory, University of València, 46100 Valencia, Spain; 5 Haemostasis and Thrombosis Unit, University and Polytechnic Hospital La Fe, 46026 Valencia, Spain

**Keywords:** haemophilia, cognitive dual-task balance, balance exercises, postural control, physical therapy

## Abstract

**Background:** Balance is an essential motor skill that enables individuals to maintain a stable posture and perform daily activities safely. Dual-task assessments are widely used to evaluate the integration of motor and cognitive functions in paediatric populations, but their effects on postural control in children with haemophilia (CwH) remain poorly understood. The objective was to analyse and compare static balance performance under single-task and cognitive dual-task conditions between CwH and age-matched healthy controls (HC). **Methods**: This comparative cross-sectional study included 34 CwH and 32 HC aged 8–12 years. Balance was assessed using the Wii Balance Board^®^ under six conditions: bipedal, bipedal with cognitive task, and dominant and non-dominant unipedal (with and without cognitive task). Primary outcome variables included overall stability index, total velocity, and sway area. Physical activity habits were also recorded in both groups with an ad hoc questionnaire. Among CwH, joint health was measured with the Haemophilia Joint Health Score (v2.0), and joint effusion/synovial hypertrophy was evaluated by ultrasound. **Results**: CwH showed significantly poorer performance in all variables, primarily under unipedal dominant/non-dominant dual-task conditions (*p* < 0.05) with percentage differences ranging from approximately 15% to over 60%. CwH reported lower-intensity and shorter-duration physical activity than HC. **Conclusions**: CwH demonstrates impaired balance, especially under unipedal and cognitive demands. Assessment of balance alongside dual-task performance may help detect subtle deficits not captured by only static balance assessment. These findings may suggest the incorporation of dual-task training into balance training programmes for this population.

## 1. Introduction

Haemophilia A and B are inherited bleeding disorders caused by deficiencies in coagulation factor VIII or IX, respectively, and classified as severe, moderate or mild according to residual factor levels [1]. Recurrent bleedings from early childhood may lead to haemophilic arthropathy, with joint degeneration, muscle weakness and impaired proprioception [2,3]. These musculoskeletal changes can compromise postural control, and children with haemophilia (CwH) often demonstrate reduced physical activity engagement and a more sedentary lifestyle, which may further affect balance-related abilities [3,4]. This is clinically relevant because structured physical activity may play a meaningful role in shaping cognitive–motor development during school age, as it provides repeated practice of coordination, timing, and goal-directed movement under varying task demands. Recent evidence shows that children with sports experience can display better fine motor coordination (e.g., manual dexterity) than sedentary peers, supporting a link between structured sport participation and motor skill development [5]. Moreover, many sport and play activities simultaneously challenge gross motor coordination and executive functions (e.g., inhibition, updating, decision-making), and cognitively engaging physical activity interventions have been associated with improvements in executive function in children and adolescents, reinforcing the tight coupling between cognitive and motor development [6]. In parallel, dual-task research indicates that maintaining stable posture and gait in typically developing children requires attentional resources, such that adding a cognitive load can alter motor control—highlighting dual-task paradigms as a useful “stress test” of cognitive–motor integration in this age range [7]. Overall, this evidence suggests that reduced participation in structured physical activity in CwH could contribute to less efficient cognitive–motor integration, potentially increasing vulnerability to balance impairments that become apparent particularly under dual-task demands.

Balance is a complex motor skill that develops progressively throughout childhood and adolescence and relies on the continuous integration of sensory, motor, and cognitive processes. During school age, postural stability is not yet fully automatized and depends on the maturation of sensory integration mechanisms (visual, vestibular, and proprioceptive systems), as well as on the development of central nervous system structures involved in motor planning and coordination [8]. Importantly, this maturation includes the progressive refinement of anticipatory and reactive postural adjustments and the ability to flexibly “reweight” sensory inputs when task or environmental constraints change, capacities that are still developing across late childhood and adolescence [9]. As a result, children require greater conscious control and attentional resources to maintain balance, particularly in challenging or dynamic conditions.

Beyond sensory and motor maturation, postural task and balance execution in children also involves developing cognitive systems. Dual-task conditions increase attentional demands and reflect the ongoing maturation of executive control (e.g., inhibitory control and working memory), such that younger children typically show greater postural interference when attentional resources must be shared across tasks [10,11]. Consistent evidence indicates that increased cognitive load can modulate balance performance in paediatric populations, supporting the view that balance control in development is partly constrained by limited attentional capacity and immature executive regulation under concurrent task demands [7,12,13].

Importantly, dual-task conditions represent an ecologically valid framework for evaluating balance in children. In daily life, children rarely perform postural tasks in isolation; instead, balance control is continuously challenged during play, social interaction, learning activities, and movement in dynamic environments. Dual-task play paradigms better reflect real-life motor behaviour by combining postural demands with concurrent cognitive activities (e.g., counting, decision-making, problem solving) allowing quantification of dual-task costs in both domains and capturing functional constraints that may be missed under single-task testing [7,10,11,12]. Therefore, assessing postural control exclusively under single-task conditions may underestimate functional impairments that become evident only when attentional resources are shared.

Evidence from paediatric research shows that dual-task interference is a characteristic of typical development, reflecting the still-maturing automatization of postural control and limited attentional resources: in typically developing children, adding a concurrent cognitive task can measurably alter postural sway and reduce stability during standing and/or locomotor tasks, with larger costs generally observed at younger ages or with higher task demands [7,14,15].

In children with neuromotor disorders, such as cerebral palsy, the addition of a cognitive task can exacerbate postural instability compared to their typically developing peers [16]. Similar patterns have also been reported in children with motor or neurodevelopmental conditions affecting coordination and sensorimotor integration (e.g., developmental coordination disorder, autism spectrum disorder, intellectual disability), suggesting a broader vulnerability of the developing cognitive–motor system under divided-attention conditions [17,18,19]. Children with other chronic paediatric conditions, such as overweight [20], show altered motor control under dual-task constraints during functional locomotor tasks, indicating that concurrent demands can worsen performance even when the primary limitation is not strictly neurological.

These findings suggest that chronic or neuromotor conditions may further compromise cognitive–motor integration during dual-task situations. However, despite this evidence in other paediatric clinical populations, studies exploring postural control under dual task conditions in CwH remain scarce, being limited to simple task paradigms [21,22] or to adult samples [23], underscoring the need for specific research in this group.

Identifying balance deficits that emerge specifically under cognitive dual-task conditions is clinically relevant because conventional single-task assessment may fail to capture instability that becomes apparent when attentional resources are divided. In our protocol, balance is challenged under ecologically meaningful conditions by combining bipedal and, especially, unipedal stance with a concurrent mental subtraction task, while quantifying postural performance through objective centre-of-pressure-derived metrics (overall stability index, total velocity, and sway area). Such metrics may remain within “normal” ranges during single-task standing yet deteriorate under dual-task loading, revealing reduced functional reserve that is directly relevant to real-life situations (e.g., play and mobility while thinking/talking), where children rarely maintain posture in isolation.

Clinically, incorporating dual-task balance testing into functional screening could improve sensitivity to detect subtle impairments in CwH, particularly in more demanding stances (unipedal dominant and non-dominant) where cognitive dual-task costs may be greatest. Combining cognitive dual-task balance metrics (e.g., total velocity or sway area) with joint evaluation by clinical and ultrasound together with reported physical activity patterns (frequency, intensity, and duration), may help stratify risk and tailor interventions: for example, counselling and graded balance training with cognitive load in children showing higher dual-task costs, joint alterations, or lower-intensity activity profiles. This approach supports rehabilitation planning that targets not only single-task stability but also cognitive–motor integration under conditions that better reflect daily participation

Therefore, the aim of this cross-sectional study is to compare static balance performance under single-task and cognitive dual-task conditions between CwH and age-matched healthy controls. We hypothesise that CwH will exhibit poorer postural stability than heathy peers under cognitive dual-task conditions.

## 2. Materials and Methods

### 2.1. Study Design

A comparative cross-sectional study was employed, attending the criteria from the Strengthening the Reporting of Observational Studies in Epidemiology (STROBE). The project received approval from the Experimental Research Ethics Committee of the University of Valencia (2142722) and adhered to the principles outlined in the Declaration of Helsinki. All participants and their legal representatives received written and verbal information prior to participation, and written consent was obtained.

### 2.2. Participants

CwH from different regions of Spain were recruited using a clinic-based convenience sampling strategy, during Haemophilia formation days and a summer camp organised by the Haemophilia Spanish Federation (Fedhemo, Madrid, Spain) in July 2022. The control group consisted of children without haemophilia recruited using a convenience sampling approach, including siblings of the recruited haemophilia children or children from the school population of Valencia. These were evaluated in a research laboratory at the University of Valencia, Valencia, Spain, between September 2022 and March 2023.

Recruitment was conducted in person through personal interviews. Eligibility criteria for the haemophilia group were: (a) age 8–12 years; (b) confirmed diagnosis of haemophilia A or B of any severity; (c) no episodes of severe knee or ankle haemarthrosis or musculoskeletal injury such as ankle sprain in the previous three months; (d) absence of any neurological condition affecting balance; and (e) basic arithmetic ability (addition and subtraction). The same criteria applied to the control group, except for the diagnosis of haemophilia.

A total of 68 children were initially assessed for eligibility. Of these, 35 children with haemophilia and 33 control group children met the inclusion criteria and were enrolled in the study. Two children declined participation. Therefore, data from a total of 66 participants (34 children with haemophilia and 32 typically developing controls) were included in the statistical analyses.

### 2.3. Outcomes Measurements

Demographic data, including age and body mass index, were collected for all participants. For CwH, the following clinical data were also recorded: type of haemophilia (A or B), severity (mild, moderate or severe), treatment modality (prophylaxis or on-demand), presence of inhibitors and bleeding episodes within the last 3 months. Additionally, physical activity patterns were assessed with a brief interview consisting of 4 questions, regarding type of exercise or sport performed throughout the year, frequency (times per week), intensity, and duration.

Joint health was assessed using the Haemophilia Joint Health Score (HJHS v2.0) and complemented by an ultrasonographic evaluation protocol. The HJHS is a standardised clinical tool designed to evaluate joint status in the elbows, knees and ankles based on nine items, which are as follows: swelling, duration of swelling, muscle atrophy, crepitus during movement, loss of flexion, loss of extension, joint pain and strength. An additional item, global gait, is scored from 0 to 4. The maximum score per joint is 20 points with a total possible score of 124 when combining all joint assessments and the gait score. Higher scores indicate poorer joint health. The HJHS has demonstrated good psychometric properties, including high internal consistency (α = 0.83–0.84) and convergent validity with the Petterson Score [24].

Joint effusion and synovial hypertrophy: In addition to the clinical evaluation, ultrasound examinations of elbows, knees and ankles were performed using an ultrasound device with a linear transducer (5–10 MHz) to assess joint effusion and synovial hypertrophy (Mindray 7L4P, Nanjing Mindray Bio-Medical Electronics Co., Nanjing, China). The ultrasound protocol included: (a) examination of the posterior olecranon recess for the elbows; (b) assessment of the suprapatellar recess in the mid-longitudinal plane, using the superior pole of the patella and quadriceps tendon insertion as anatomical landmarks; and (c) examination of the anterior recess of the tibiotalar joint for the ankles [25,26]. All clinical and ultrasonographic assessments were conducted by a physiotherapist with over five years of experience in the management of patients with haemophilia.

**Figure 1:**
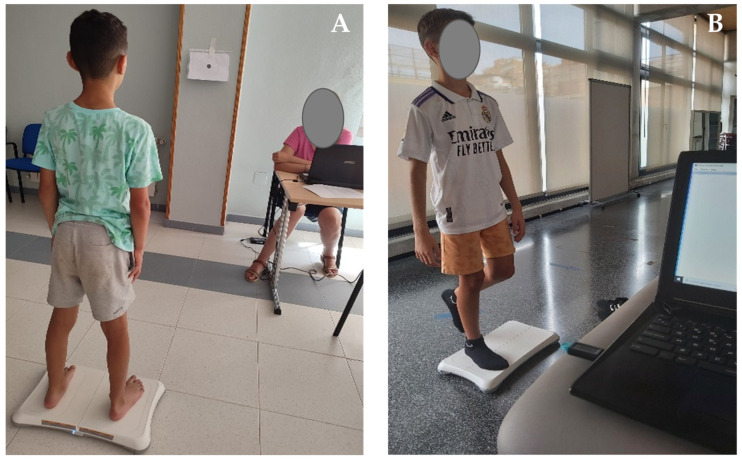
Balance assessment. (**A**) Bipedal test during dual task in children with haemophilia. (**B**) Unipedal balance during dual task in a child from control group.

Balance is the primary outcome of this study. It was assessed using the Wii Balance Board^®^ (Nintendo Co., Ltd., Kyoto, Japan), which is a reliable and valid tool for measuring the centre of pressure analysed in several studies [27,28,29]. We apply both simple and cognitive dual-task conditions, in bipedal and unipedal support. Before starting the evaluation, the different test conditions were explained to each subject, and the dominant leg of each participant was determined. The dominance test consisted of throwing a ball to the child at ground level, then noting with which leg he performed the kick. They were instructed to maintain an upright standing posture for 30 s during each test. For each trial, a target was placed at eye level, and the children were instructed to keep their eyes fixed at the target’s mid-point. The cognitive dual task consisted of maintaining balance while performing subtraction by threes aloud, starting from a different number in each test (100 being the highest number they started with), in order to prevent children from memorising the sequence. This was standardised for all children, and the order of the tests was randomised to control for potential learning or training effects that could arise from repeated exposure to similar tasks. The BrainBlox programme (University of Colorado, Boulder, CO, USA) was used to synchronise and record the data for each trial. Data were sampled at a frequency of 80 Hz and subsequently filtered using a sixth-order Butterworth low-pass filter with a 12 Hz cut-off frequency. The following tests were performed [30]: (a) bipedal balance simple task (BB), (b) bipedal balance with a DT (BB-DT); (c) unipedal balance on the dominant leg (UBD); (d) UBD with a DT (UBD-DT); (e) unipedal balance on the non-dominant leg (UBND); and (f) UBND with a DT (UBND-DT) (Figure 1). The variables analysed were as follows: overall stability index (OSI, cm); total velocity (TV, cm/s); and sway area (SA, cm^2^). Lower values in all variables indicate greater postural stability and consequently, better balance performance.

### 2.4. Data Analysis

All analyses were performed using SPSS Statistics software for Windows (Version 26.0; IBM Corp, Armonk, NY, USA). The Shapiro–Wilk test was used to assess the normality of continuous variables. Descriptive statistics were reported as mean (standard deviation), median [25–75th percentile], or frequency (percentage), as appropriate.

Linear mixed-effects models (LMMs) were fitted for each dependent variable (OSI, MTV, and SA). Group (CwH vs. HC), Task (simple, dual task), and Condition (BB, UBD, and UBND) were included as fixed effects, along with all interactions. A random intercept for participants was included to account for within-subject dependence. The repeated-measures covariance structure for Task and Condition was modelled using an ARH1 structure. Models were estimated using REML, and denominator degrees of freedom were calculated using the Satterthwaite approximation. Outcome variables were log-transformed using the natural logarithm to improve normality and homoscedasticity.

Type III tests of fixed effects were used to evaluate main effects and interactions. When significant effects were detected, pairwise comparisons of estimated marginal means were performed with Bonferroni adjustment for multiple testing. Statistical significance was set at *p* < 0.05.

Because the dependent variables were analysed on the log-transformed scale, between-group differences from pairwise comparisons were back-transformed and expressed as percentage difference ratios to facilitate interpretation on the original scale.

Effect sizes for fixed effects were quantified using partial eta squared (ηp^2^) derived from the F-statistics of the mixed models. Values of 0.01, 0.06, and 0.14 are commonly interpreted as small, medium, and large effects, respectively [31].

Custom algorithms developed in MATLAB (version R2018b; The MathWorks Inc., Natick, MA, USA) were used to process the Wii Balance Board^®^ signals and compute the stability indices.

### 2.5. Sample Size

An a priori power analysis was conducted using G*Power 3.1.9.2 software (Heinrich-Heine-Universität, Düsseldorf, Germany) to determine the required sample size. Based on the study design, with an alpha level of 5% (α = 0.05), a desired power of 80% and at least a medium effect size (ηp^2^ ≈ 0.06) expected for group differences in postural control, a minimum of 30 participants per group was estimated.

The expected effect size was derived from preliminary data in a previous study assessing postural control variables (e.g., OSI, SA) in CwH using comparable balance tasks [29].

## 3. Results

### 3.1. Demographic and Clinical Data

Table 1 shows the descriptive characteristics of the participants. No significant differences were observed between the two groups in age, body mass, height or BMI.

Table 2 presents the clinical variables of the CwH. Of the 34 participants, 32 had haemophilia A and 2 had haemophilia B. The majority had severe haemophilia (n = 28), while 5 had moderate and 1 had mild haemophilia. All participants, except one, were receiving prophylactic treatment, and 4 children had a history of inhibitors. Two participants reported a single bleeding episode in the elbow within the past 3 months.

Regarding clinical joint health, assessed using the Haemophilia Joint Health Score (HJHS, v. 2.0), 21 (61.76%) participants showed no joint alterations. However, 13 (38.23%) children presented joint changes: 9 children in the knees (min. 1–max. 3 points), 10 in the ankles (min. 1–max. 2 points) and 5 in the elbows (min. 1–max. 10 points). Ultrasound imaging revealed increased synovial hypertrophy in 4 elbows, 7 knees and 13 ankles without associated effusion (Table 2).

### 3.2. Physical Exercise Patterns

Among CwH, 11.8% reported not engaging in any physical exercise, compared to only 3% in the healthy control group. Conversely, the remaining participants in both groups reported participating in some form of physical activity or sport as part of their daily routine (Table 3). No significant differences were observed between groups in terms of exercise frequency with 2–3 days per week being the most common, reported by 65.5% of healthy children and 44.1% of CwH. However, significant differences were found in exercise intensity: 20.9% of CwH reported engaging in low-intensity exercise, whereas 93.8% of healthy children reported participating in high intensity exercise.

Similarly, significant group differences were observed in exercise duration. The most common duration among CwH was 30 to 60 min (44%), while 62.5% of healthy participants exercised for more than one hour. Notably, some CwH exercised for less than 15 min (5.9%) or between 15 and 30 min (14.7%), while no participants in the healthy group reported exercising for less than 30 min, with 34.4% exercising for 30 to 60 min.

### 3.3. Balance

Linear mixed models revealed significant main effects of the Group for OSI (*F *(1, 58.3) = 6.3, *p* = 0.015, moderate effect size), TV (*F* (1, 49.9) = 5.9, *p* = 0.018, moderate effect), and SA (*F* (1, 50.9) = 8.0, *p* = 0.007, ηp^2^ = 0.136, large effect), indicating overall poorer balance in CwH compared with HC. A significant main effect of Task was also observed for TV (*F* (1, 119.4) = 16.2, *p* < 0.001, moderate effect size) and SA (*F* (1, 138.0) = 4.7, *p* = 0.032, small effect), indicating that dual-task performance generally increased postural instability compared with single-task performance (Table 4).

Significant main effects of Condition were found across all variables with large effect sizes, reflecting increased postural instability from bipedal to monopedal conditions. Significant Task × Condition interactions were observed for the 3 variables, indicating that the effect of dual-tasking was stronger in monopedal than in bipedal conditions. Smaller but relevant interactions (e.g., Group × Task, Group × Condition) suggested that dual-tasking amplified between-group differences in specific conditions.

Under the simple (single-task) condition, no significant between-group differences were observed in either the bipedal tests or in the unipedal balance of the non-dominant leg (*p* > 0.05). In contrast, in the unipedal dominant leg condition, CwH exhibited higher values than HC, indicating poorer balance. Percentage differences in this condition were 19.6% for OSI, 20.1% for MTV, and 44.6% for SA (Table 5).

In the dual-task condition, group differences were more pronounced across unipedal tests. CwH consistently exhibited higher values than HC, reflecting greater postural instability under cognitive load. Percentage differences ranged from 16.4% to 63.2%, depending on the variable and leg. These findings indicate that performing a dual task further exacerbates postural instability in CwH.

## 4. Discussion

To our knowledge, this is the first comparative cross-sectional study to analyse cognitive dual-task balance in children with haemophilia (CwH) versus healthy controls (HC).

Based on the results, the hypothesis was partially confirmed. CwH exhibited poorer balance overall compared with healthy peers, but significant differences were observed primarily during the cognitive dual-task condition in unipedal tests. During the dual-task condition, CwH consistently showed higher values than HC, with percentage differences ranging from approximately 15% to over 60% depending on the variable and leg, indicating that performing a cognitive dual-task further exacerbates postural instability in this population. These findings align with prior reports of motor-control deficits in this population and suggest greater reliance on compensatory postural strategies [32,33,34].

Our results also underscore the cognitive burden inherent to dual-tasking in CwH. Engaging attentional resources while maintaining posture can degrade balance performance in groups with physical constraints [33,35], and everyday activities frequently impose concurrent cognitive–motor demands [34,36]. In paediatric samples, adding a cognitive task alters sway patterns and worsens stability [37,38], which is consistent with our observation that cognitive load amplifies existing balance deficits in CwH. Because this study is cross-sectional, causal inferences cannot be drawn; longitudinal designs are needed to track how balance and cognitive–motor integration evolves over time.

From a rehabilitation perspective, the data support task-specific interventions that integrate cognitive dual-task elements—especially in unipedal or otherwise challenging stances. Evidence from other populations (e.g., cerebral palsy, older adults, chronic stroke) indicates that dual-task training can enhance balance and cognitive–motor coupling [39,40], which is likely relevant to CwH. To date, only one trial in haemophilia (adults with arthropathy) has incorporated cognitive dual-task balance within a multimodal programme, improving balance, function, fall risk and strength, although dual-task outcomes were limited to bipedal stance and showed no significant change [41].

Future studies in paediatric CwH should test progressive dual-task balance protocols (e.g., unipedal/unstable conditions, graded cognitive loads) and evaluate both postural and cognitive outcomes [42,43,44].

Clinical context remains important. Recurrent joint bleeding may lead to synovial hypertrophy, pain, reduced range of motion and weakness, all of which can impair balance and restrict physical activity. In our sample, ultrasound revealed ankle synovial hypertrophy in 13 participants, which may have contributed to poorer performance [2,34].

Previous research shows substantial variability in physical activity levels among CwH, which is often associated with reduced joint stability and strength due to lower exercise engagement compared with HC [45,46]. Regular participation in physical activity has been associated with improvements in joint health and proprioception, both essential for postural control [47]. In our sample, exercise frequency did not differ significantly between groups; however, clear differences emerged in intensity and duration. Healthy children engaged in higher-intensity and longer-duration activities, consistent with reports that CwH may avoid vigorous exercise because of bleeding concerns [48,49]. Beyond physical benefits, regular activity also supports psychosocial well-being—an area where CwH frequently face challenges [50]. These findings reinforce the need for tailored exercise programmes that promote safe, appropriate activity levels while accounting for the specific health needs of this population.

This study has limitations. There may be selection bias related to recruitment in clinics and convenience, which may limit the generalizability of the results. The cognitive dual-task assessment consisted solely of a subtraction task. Including different types of cognitive tasks would have allowed for a better exploration of the differential impact of cognitive demands on postural control. Moreover, exercise habits were interview-based rather than measured with validated tools (e.g., IPAQ) or accelerometry, reducing comparability. In addition, balance was assessed using the Wii Balance Board, a cost-effective tool with acceptable validity for static posturography. However, as a consumer-grade device, it provides lower sampling resolution than laboratory-grade force platforms, which may limit its sensitivity to subtle or dynamic postural adjustments. Therefore, the results should be interpreted within the context of static balance tasks and the specific parameters analysed.

Also, this study has several strengths that should be highlighted. First, to our knowledge, it is the first study to systematically assess static balance under cognitively demanding dual-task conditions in CwH, addressing an important gap in the paediatric haemophilia literature. Second, the combination of unipedal stance with a concurrent cognitive task represents an ecologically valid challenge that closely resembles everyday situations in which children must manage balance while simultaneously engaging in cognitive activities. Third, the integration of balance outcomes with joint health scores, ultrasound findings, and physical activity patterns provides a comprehensive clinical perspective that may support more individualised assessment and rehabilitation strategies in children with haemophilia.

## 5. Conclusions

Children with haemophilia showed balance impairments—most pronounced under unipedal and cognitive dual-task conditions—highlighting the interplay between cognitive load and postural control. These deficits may be compounded by the typically lower intensity and duration of physical activity in this group. Our findings support implementing comprehensive training programmes that address both physical and cognitive components (e.g., balance and strength work with graded dual-task challenges) to improve stability and enhance functionality in CwH.

## Figures and Tables

**Table 1:** Demographic variables.

	CwH (*n* = 34) Mean (SD) or Median [IQR]	HC (n = 32) Mean (SD) or Median [IQR]	Intra-Group Analysis *p* [95% CI] or *p*; z
Age (years)	10.00 [9.00–11.25]	10.00 [9.00–12.00]	0.87; −0.15
Height (m)	1.42 (0.11)	1.42 (0.13)	0.75 [−0.07–0.05]
Body mass (kg)	38.25 [29.75–46.55]	36.5 [32–38.75]	0.49; −0.69
BMI (kg/m^2^)	18.47 [16.71–21.11]	17.58 [16.64–19.37]	0.21; −1.26

Data are shown with mean (standard deviation) or median (interquartile range: 25–75 percentile) unless otherwise indicated. Results of the analyses are shown with *p* (95% CI of the mean difference) for the *t*-test or with *p*; z test for the Mann–Whitney U test. SD: Standard deviation; IQR: Interquartile range; BMI: Body mass index; CwH: Children with haemophilia; and HC: Healthy controls.

**Table 2:** Clinical variables for haemophilia children (n = 34).

	CwH
Type haemophilia (A/B)	32/2
Severity (mild/moderate/severe)	1/5/28
Treatment (prophylaxis/on demand)	33/1
Inhibitors (No/Yes)	30/4
Bleedings in last 3 months (No/Yes)	32/2
Total HJHS (0/1/2/3/10/11/12)	21/2/6/2/1/1/1
HJHS Right Elbow (0/1/5/10)	31/1/1/1
HJHS Left Elbow (0/3/4)	32/1/1
HJHS Right Knee (0/1/2)	29/4/1
HJHS Left Knee (0/1/2/3)	30/1/2/1
HJHS Right Ankle (0/1/2)	30/3/1
HJHS Left Ankle (0/1/2)	28/1/5
HJHS Global gait (0)	34
SYNOVIAL HYPERTROPHY (No/Yes)	
Synovial hypertrophy Right Elbow	32/2
Synovial hypertrophy Left Elbow	32/2
Synovial hypertrophy Right Knee	31/3
Synovial hypertrophy Left Knee	30/4
Synovial hypertrophy Right Ankle	28/6
Synovial hypertrophy Left Ankle	27/7
Effusion Right Elbow	34/0
Effusion Left Elbow	34/0
Effusion Right Knee	34/0
Effusion Left Knee	34/0
Effusion Right Ankle	34/0
Effusion Left Ankle	34/0

Frequencies have been used. CwH: Children with haemophilia; and HJHS: Haemophilia Joint Health Score.

**Table 3:** Type, frequency, intensity and time of exercises or sport.

	CwH n (%)	HC n (%)	Chi2 (*p*)
Physical exercise or sport			
0. Nothing	4 (11.8%)	1 (3.1%)	-
1. Football	7 (20.6%)	22 (68.8%)	
2. Swimming	8 (23.5%)	2 (6.3%)	
3. Bicycle	2 (5.9%)	0	
4. Walk	3 (8.8%)	0	
5. Tennis	4 (11.8%)	1 (3.1%)	
6. Athletics	2 (5.9%)	0	
7. Run	1 (2.9%)	1 (3.1%)	
8. Handball	1 (2.9%)	1 (3.1%)	
9. Volleyball	0	1 (3.1%)	
10. Padel	0	2 (6.3%)	
11. Basketball	2 (5.9%)	1 (3.1%)	
Exercise frequency			
0. Very sporadically	1 (2.9%)	0	3.83 (0.28)
1. Once a week	4 (11.8%)	1 (3.1%)	
2. 2–3 times a week	15 (44.1%)	21 (65.5%)	
3. Almost every day	10 (29.4%)	9 (25.0%)	
***Intensity***			
1. Soft	7 (20.6%)	0	14.15 (0.001)
2. Hard	17 (50.1%)	30 (93.8%)	
3. Exhausted	6 (17.0%)	1 (3.1%)	
***Duration of exercise session***			
0. <15 min	2 (5.9%)	0	12.74 (0.005)
1. Between 15 and 30 min	5 (14.7%)	0	
2. between 30 and 60 min	15 (44.0%)	11 (34.4%)	
3. More than 1 h	8 (23.1%)	20 (62.5%)	

Data are shown with the number of subjects (n) and percentage (%). Results of the analyses are shown with chi-squared (p) values. CwH: Children with haemophilia; HC: Healthy controls.

**Table 4:** Type III tests of fixed effects for Group, Task, and Condition in OSI, MTV, and SA stability indices.

	OSI			TV			SA		
Effect	F (ndf, ddf)	*p*	ηp^2^	F (ndf, ddf)	*p*	ηp^2^	F (ndf, ddf)	*p*	ηp^2^
Group	6.3 (1, 58.3)	**0.015**	0.10	5.9 (1, 49.9)	**0.018**	0.11	8.0 (1, 50.9)	**0.007**	0.14
Task	0.8 (1, 138.8)	0.38	-	16.2 (1, 119.4)	**<0.001**	0.12	4.7 (1, 138.0)	**0.032**	0.03
Condition	80.6 (2, 148.6)	**<0.001**	0.52	666.0 (2, 139.1)	**<0.001**	0.91	345.8 (2, 145.3)	**<0.001**	0.83
Group × Task	1.4 (1, 138.8)	0.24	-	3.6 (1, 119.4)	0.06	-	4.3 (1, 138.0)	**0.039**	0.03
Group × Condition	1.2 (2, 148.6)	0.29	-	4.4 (2, 139.1)	**0.014**	0.06	1.3 (2, 145.3)	0.28	-
Task × Condition	4.9 (2, 97.1)	**0.010**	0.09	9.9 (2, 98.2)	**<0.001**	0.17	8.9 (2, 95.4)	**<0.001**	0.16
Group × Task × Condition	0.6 (2, 297.1)	0.53	-	0.7 (2, 98.2)	0.50	-	1.22 (2, 95.4)	0.30	-

F: F-statistic; *p*: significance level; ηp^2^: partial eta squared; OSI: Overall Stability Index; TV: Total Velocity; and SA: Sway Area. df: Degrees of freedom are shown in parentheses as (numerator df, denominator df). Significant results are highlighted in bold.

**Table 5:** Percentage differences between CwH and HC in OSI, TV, and SA stability indices across tasks and conditions.

		OSI		TV		SA	
Task	Condition	% Diff [95% CI]	*p*	% Diff [95% CI]	*p*	% Diff [95% CI]	*p*
Simple	BB	5.9 [−15.2, 31.9]	0.61	0.2 [−13.8, 16.3]	0.98	6.3 [−23.0, 46.8]	0.71
	UBD	19.6 [5.0, 36.0]	**0.008**	20.1 [2.8, 40.2]	**0.022**	44.6 [10.2, 89.8]	**0.008**
	UBND	4.1 [−11.6, 22.4]	0.63	17.4 [−0.5, 38.5]	0.06	15.9 [−15.2, 58.3]	0.35
Dual	BB	19.0 [−9.0, 55.5]	0.20	9.8 [−12.6, 38.1]	0.42	35.1 [−12.6, 109.0]	0.17
	UBD	19.4 [6.8, 33.4]	**0.002**	24.2 [7.5, 43.6]	**0.004**	51.5 [20.6, 90.8]	**<0.001**
	UBND	16.4 [2.6, 32.0]	**0.019**	35.7 [16.8, 57.7]	**<0.001**	63.2 [28.5, 107.3]	**<0.001**

Values are calculated from log-transformed data. % difference = (e^mean^ ^difference^ − 1) × 100. Positive values indicate that the stability index is higher in the CwH group than in the HC group. Confidence intervals for the mean differences are 95% Bonferroni-corrected. Significant comparisons (*p* < 0.05) are highlighted in bold. CwH: Children with Haemophilia; HC: Healthy Controls; OSI: Overall Stability Index; TV: Total Velocity; SA: Sway Area; BB: Bipedal balance; UBD: Unipedal balance dominant; and UBND: Unipedal balance non-dominant. Significant results are highlighted in bold.

## Data Availability

The original contributions presented in this study are included in the article/Supplementary Materials. Further inquiries can be directed to the corresponding author.

## Supplementary Materials

The following supporting information can be downloaded at: https://www.mdpi.com/article/10.3390/jfmk11010067/s1.

## Abbreviations

The following abbreviations are used in this manuscript:

CwH	Children with Haemophilia
HJHS	Haemophilia Joint Health Score
BB	Bipedal Balance
UBD	Unipedal Balance Dominance
UBND	Unipedal Balance Non-Dominance
DT	Dual Task
