# Supplementary material for: Assessment of Static Balance with and Without Cognitive Dual Task in Children with Haemophilia: A Cross-Sectional Study
Source: J Funct Morphol Kinesiol. 2026 Feb 6;11(1):67. doi: 10.3390/jfmk11010067 (PMC12921909; doi:10.3390/jfmk11010067)
Supplement: Supplementary file 1 [file jfmk-11-00067-s001.zip › File S1. README.pdf]

README – Minimal Dataset Description

General Description

This dataset corresponds to the minimal data required to reproduce the statistical analyses reported in the associated manuscript submitted to MDPI. The study investigates balance performance in children with haemophilia assessed using the Nintendo Wii Balance Board.

The dataset is provided in Microsoft Excel format and is ready to be imported into standard statistical software (e.g., SPSS, R, or similar).

Confidential, proprietary and personally identifiable information has been removed.

Data Processing

Balance-related variables were calculated from the Wii Balance Board signals using a custom MATLAB script. These variables represent commonly used indices of postural control. As the aim of this dataset is to enable replication of the statistical analyses, only the processed outcome variables are included.

Dataset Structure

- **File format:** Microsoft Excel (.xlsx).
- **Sheet "Data" (primary data):**
    - **Rows:** Individual participants / trials.
    - **Columns:** Balance indices.
- **Sheet "Description" (metadata):**
    - Variable name.
    - Description.
    - Measurement units.

Outliers

Outliers were identified and removed **separately for each test/condition**.

Missing Data

Empty cells in the dataset indicate **missing data**, which may be due to incomplete or invalid trials. No imputation was performed on missing values.

Data Usage

The dataset can be directly used for:

- Descriptive statistics.
- Inferential statistical analyses.
- Replication of the results reported in the manuscript.

Related Publication

This dataset is associated with the following manuscript:

"Assessment of static balance with and without cognitive dual task in children with haemophilia: a cross-sectional study "

Contact

For questions regarding the dataset or data processing procedures, please contact the corresponding author.
